# Supplementary material for: Heat adaptation of phage T7 under an extended genetic code
Source: Virus Evol. 2021 Dec 1;7(2):veab100. doi: 10.1093/ve/veab100 (PMC8923235; doi:10.1093/ve/veab100)
Supplement: veab100_Supp [file veab100_supp.zip › Sequencing_Analysis.zip!/Sequencing_Analysis/A2/evidence/RA_25.html]

BRESEQ :: Evidence


| Read alignment evidence... | | | | | | | | | | | |
| --- | --- | --- | --- | --- | --- | --- | --- | --- | --- | --- | --- |
|  | seq id | position | | ref | new | freq | score (cons/poly) | reads | annotation | genes | product |
| \* | NC\_001604.1 | 27,783 | 0 | G | A | 67.7% | 397.2 / inf | 510 | intergenic (–/–) | *–/–* | –/– |
| Reads supporting (aligned to +/- strand):  **ref** base G (81/84);  **new** base A (171/174);  **total** (252/258) | | | | | | | | | | | |
| Fisher's exact test for biased strand distribution *p*-value = 9.25e-01 | | | | | | | | | | | |
| Kolmogorov-Smirnov test that lower quality scores support variant *p*-value = 5.28e-01 | | | | | | | | | | | |

Only 100 of 511 total aligned reads displayed.  

`GCTGGTTCGTTACGAGCGACCAAGTGTGGCGACTTAGTGGAAAGGCTAAGCGAAAGTTCCGTAAGTTAATCATGGAGTATCGCGATAAGATGCTTGAGAAGTATGATACTCTTTGGAATTACGTATGGGTAGGCAATACGTCCCACATTCGTTTCCTCAAGACTATCGGTGCGGTATTCCATGAAGAGTACACACGAGATGGTCAATTTCAGTTATTTACAATCACGAAAGGAGGATAACCATATGTGTTGGGCAGCCGCAATACCTATCGCTATATCTGGCGCTCAGGCTATCAGTGGTCAGAACGCTCAGGCCAAAATGATTGCCGCTCAGACCGCTGCTGGTCGTCGTCAAGCTATGGAAATCATGAGGCAGACGAACATCCAGAATGCTGACCTATCGTTGCAAGCTCGAAGTAAACTTGAGGAAGCGTCCGCCGAGTTGACCTCACAGAACATGCAGAAGGTCCAAGCTATTGGGTCTATCCGAGCGGCTATCGGAGAGAGTATGCTTGAAGGTTCCTCAATGGACCGCATTAAGCGAGTCACAGAAGGACAGTTCATTCGGGAAGCCAATATGGTAACTGAGAACTATCG  >  NC_001604.1/27485‑28080`  

                                                                                                                                                                                                                                                                                                          `|`                                                                                                                                                                                                                                                                                                           
                  `aCCAAGTGTGGCGACTTAGTGGAAAGGCTAAGCGAAAGTTCCGTAAGTTAATCATGGAGTATCGCGATAAGATGCTTGATAAGTATGATACTATTTGGAATTACGTATGGGTAGGCAATACGTCCCACATTCGTTTCCTCAAGACTATCGGTGCGGTATTCCATGAAGAGTACACACGAGATGGTCAATTTAAGTTATTTACAATCACGAAAGGCGGATAACCCTATGTGTTGGGCAGCCGCAATGCCTATCGCTATATCTGGCGCTCAGGATATCAGTGATCAGAACGATCAGGCCaaca                                                                                                                                                                                                                                                                                       >  1:386050/1‑299 (MQ=255)`  
                                   `aagcgaacgttccgtaagttaatcatggagtatcgcgataagatgcttgagaagtatgatactctttggaattacgtatgggtaggcaatacgtcccacattcgtttcctcaagactatcggtgcggtattccatgaagagtacacacgagatggtcaatttcagttatttacaatcacgaaaggaggataaccatatgtgttgggcagccgcaatacctatcgctatatctggCGCTATATCTGGCGCTCAGGCTATCAGTTGTCAGAACGCTCAGGCCAAAATGATTGCCGCTCAGAcc                                                                                                                                                                                                                                                                      <  1:143457‑M2/67‑1 (MQ=255)`  
                                    `gtgtAAAGGCTAAGCGAAAGTTCCGTAAGTTAATCATGGAGTATCGCGATAAGATGCTTGAGAAGTATGATACTCTTTGGAATTACGTATGGGTAGGCAATACGTCCCACATTCGTTTCCTCAAGACTATCGGTGCGGTATTCCATGAAGAGTACACACGAGATGGTCAATTTCAGTTATTTACAATCACGAAAGGAGGATAACCATATGTGTTGGGCAGCCGCAATACCTATCGCTATATCTGGCGCTCAGGCTATCAGTGATCAGAACGCTAAGGCCAAAATGATTGCCGCTCAGAcca                                                                                                                                                                                                                                                                     >  1:535385/1‑300 (MQ=255)`  
                                    `gTGGAAAGGCTAAGCGAAAGTTCCGTAAGTTAATCATGGAGTATCGCGATAAGATGCTTGAGAAGTATGATACTCTTTGGAATTACGTATGGGTAGGCAATACGTCCCACATTCGTTTCCTCAAGACTATCGGTGCGGTATTCCATGAAGAGTACACACGAGATGGTCAATTTCAGTTATTTACAATCACGAAAGGAGGATAACCATATGTGTTGGGCAGCCGCAATACCTATCGCTATATCTGGCGATCAGGCTATCAGTGATCAGAACGCTCAGGCCAACATGAtctgtctcttataca                                                                                                                                                                                                                                                                     >  1:673719/1‑287 (MQ=255)`  
                                        `agagacagaGCGAAAGTTCCGTAAGTTAATCATGGAGTATCGCGATAAGATGCTTGAGAAGTATGATACTCTTTGGAATTACGTATGGGTAGGCAATACGTCCCACATTCGTTTCCTCAAGACTATCGGTGCGGTATTCCATGAAGAGTACACACGAGATGGTCAATTTCAGTTATTTACAATCACTAAAGGAGGATAACCATATGTGTTGGGCAGCCGCAATACCTATCGCTATATCTGGCGCTCAGGCTATCAGTGATCAGAACGCTCAGGCCAAAATGATTGCCGCTCAGACCgctgc                                                                                                                                                                                                                                                                 <  1:549937/293‑1 (MQ=255)`  
                                           `ggCTAAGCGAAAGTTCCGTAAGTTAATCATGGAGTATCGCGATAAGATGCTTGAGAAGTATGATACTCTTTGGAATTACGTATGGGTAGGCAATACGTCCCACATTCGTTTCCTCAAGACTATCGGTGCGGTATTCCATGAAGAGTACACACGAGATGGTCAATTTCAGTTATTTACAATCACGAAAGGAGGATAACCATATGTGTTTGGCAGCCGCAATACCTATCGCTATATCTGGCGCTCAGGCTATCAGTGATCAGAACGCTCAGGCCAAAATGATTGCCGCTCAGACCGCTGCTgg                                                                                                                                                                                                                                                              >  1:761368/1‑301 (MQ=255)`  
                                                    `gttaatcatggagtatcgcgataagatgctttagaagtatgatactctttggaattacgtatgggtaggcaatacgtcccacattcgtttcctcaagactaacggtgcggtattccatgaagagtacacacgagatggtcaatatcagttatttacaatcacgaaaggaggatatccatatgtgttgggcagccgcaatactgatcgctatatctggCGCTATATCTGGCGCTCAGGCTATCAGTGGTCAGAACGCTCAGTCCAAAATGATTGCCGCTCAGaccgctgctggctgtctctt                                                                                                                                                                                                                                                     >  1:50416‑M2/218‑292 (MQ=255)`  
                                                       `gtataagagacagaTCATGGAGTATCGCGATAAGATGCTTGATAAGTATGATACTTTTTGGAATTACGTATGGGTAGGAAATACGTCCCACATGCGTTTCCTCAAGACTATCGGTGCGGTATTCCATGAAGAGTACACACGAGATGGTCAATTTCAGTTATTTACAATCACGAAAGGAGGATAACCATATGTGTTGGGCAGCCGCAATACCTATCGCTATATCTTGCGCTCAGGCTATCAGTGATCAGAACGCTCAGGCCAAAATGATTGCCGCTCAGACCGCTGCTGGTCGTCGTCAAGc                                                                                                                                                                                                                                                  <  1:566408/288‑1 (MQ=255)`  
                                                           `atggagtatcgcgataagatgcttgagaagtatgatactctttggaattacgtatgggtaggcaatacgtcccacattcgtttcctcaagactatcggtgcggtattccatgaagagtacacacgagatggtcaatttcagttatttacaatcacgaaaggaggataaccatatgtgttgggcagccgcaatacctatcgctatatctggCGCTATATCTGGCGCTCAGTCTATCAGTGGTCAGAATGCTCAGGCCAAAATGATTGCCGCTCAGACCGCTGCTGGTCGTCGTCAAGCTATg                                                                                                                                                                                                                                              >  1:578935‑M2/211‑301 (MQ=255)`  
                                                                   `tcgcgataagatgcttgagaagtatgatactctttggaattacgtatgggtaggcaatacgtcccacattcgtttcctcaagactatcggtgcggtattccatgaagagtacacacgagatggtcaatttcagttatttacaatcacgaaaggaggataaccatatgtgttgggcagccgcaatacctatcgctatatctggCGCTATATCTGGCGCTCAGGCTATCAGTGGTCAGAACGCTCAGGCCAAAATGATTGCCgctcagaccgctgctggtcatcgtctgtctcttatacacat                                                                                                                                                                                                                                      >  1:601072‑M2/203‑285 (MQ=255)`  
                                                                       `ctGGAGTATCGCGATAAGATGCTTGAGACGTATGATACTCTTTTGAATTACGTATTGGTAGGCAATACGTCCCACATTCGTTTCCTCAAGACTATCGGTGCTGTATTCCATGAAGAGTACCCACGCGATGGTCAATTTCAGTTATTTACAATCACGAAAGGAGGATAACCATATGTGTTGGGCAGCCGCAATACCTATCGCTATATCTGGCGCTCAGGCTATCAGTTATCAGAACGCTCAGGCCAAAATGATTGCCGCTCAGACCGCTGCTGGTCGTCGTCAAGCTATGGAAATCATGAgg                                                                                                                                                                                                                                  <  1:785364/300‑1 (MQ=255)`  
                                                                        `tgtaGTATCGCGATAAGATGCTTGAGAAGTATGAAACTCTTTGGAATTACGTATGGGTAGGCAATACGTCCCACATTCGTTTCCTCAAGACTATCGGTGCGGTATTCCATGAAGAGTACACACGAGATGGTCAATTTCAGTTATTTACAATCACGAAAGGAGGATAACCATATGTGTTGGGCAGCCGCAATACCTATCGCTATATCTGGCGCTCAGGCTATCAGTGATCAGAACGCTCAGGCCAAAATGATTGCCGCTCAGACCGCTGCTGGTCGTCGTCAAGCTATGGAAATCATGAGGc                                                                                                                                                                                                                                 <  1:585989/298‑1 (MQ=255)`  
                                                                              `aTCGCGATAAGATGCTTGAGAAGTATGATACTCTTTGGAATTACGTATGGGTAGGCAATACGTCCCACATTCGTTTCCTCAAGTCTATCGGTGCGGTATTCCATGAAGAGTACACACGAGATGGTCAATTTCAGTTATTTACAATCACGAAAGGAGGATAACCATATGTGTTGGGCAGCCGCAATACCTATCGCTATATCTGGCGCTCAGGCTATCAGTGATCAGAACGCTCAGGCCAAAATGATTGCCGCTCAGACCGCTGCTGGTCGTCGTCAAGCTATGGAAATCATGAGGCatgtct                                                                                                                                                                                                                           >  1:813727/1‑296 (MQ=255)`  
                                                                                      `gggagatggggataagggaccgctgtttGGAATTACGTAGGGGTAGGCACTACGACCCACATTCGTTTCCTCAAGACTATCGGTGCGGTATTCCATGAAGAGTACACACGAGATGGTCAATTTCAGTTATTTACAATCACGAAAGGAGGATAACCATATGTGTTGGGCAGCCGCAATACCTATCGCTATATCTGGCGCTCAGGCTATCAGTGATCAGAACGCTCAGGCCAAAATGATTGCCGCTCAGACCGCGGCTGGTCGTAGTCAAGCTATGGAAATCATGAGGCAGACGAACATCCAg                                                                                                                                                                                                                   <  1:797081/276‑1 (MQ=255)`  
                                                                                           `gCTTGAGAAGTATGATACTCGTTGGAATTACGGATGGGTAGGCAATACGTCCCACATTCGTTTCCTCAAGACTATCGGTGCGGTATTACATGAAGAGTACACACGAGATGGTCAATTTCAGTTATTTACAATCACGAAAGGAGGATAACCATATGTGTTGGGAAGCCGCAATACCTATCGCTATATCTGGCGCTCAGGCTATCAGTGATCAGAACGCTCAGGCCAAAATGATTGCCGCTCAGACCGCTGCTGGTCGTCGTCAAGCTATGGAAATCATGAGGCAGACGAACATCCAGAATGc                                                                                                                                                                                                              <  1:109956/301‑1 (MQ=255)`  
                                                                                                  `ctttggaattacgtatgggtaggcaatacgtcccacattcgtttcatcaagactatcggtgcggtattccatgaagagtacacacgagatggtcaatttcagttatttacaatcacgaaaggaggataaccatatgtgttgggcagccgcaatacctatcgctatatctggCGCTATATCTGGCGCTCAGGCTATCAGTGGTCAGAACGCTCAGGCCAAAATGATTGCAGCTCAGACCGCTGCTGGTCGTCGTCAAGCTATGGAAATCATGAGGCCGACGAACATCCAGACTGCTGACCTa                                                                                                                                                                                                       >  1:634653‑M2/172‑301 (MQ=255)`  
                                                                                                    `gTATGATACTCTTTGGAATTACGTATGGGTAGGCAATACGTCCCACATTCGTTTCCTCAAGACTATCGGTGCGGTATTCCATGAAGAGTACACACGAGATGGTCAATTTCAGTTATTTACAATCACGAAAGGAGGATAACCATATGTGTTGGGCAGCCGCAATACCTATCGCTATATCTGGCGCTCAGGCTATCAGTGATCAGAACGCTCAGGCCAAAATGATTGCCGCTCAGACCGCTGCTGGTCGTAGTCAAGCTATGGAAATCATGAGGCAGACGAACATCCAGAATGCTGACCTATc                                                                                                                                                                                                     >  1:140692/1‑301 (MQ=255)`  
                                                                                                      `aTGATACTCTTTGGAATTACGTATGGGTAGGCAATACGTCCCACATTCGTTTCCTCAAGACTATCGGTGCGGTATTCCATGAAGAGTACACACGAGATGGTCAATTTCAGTTATTTACAATCACGAAAGGAGGATAACCATATGTGTTGGGCAGCCGCAATACCTATCGCTATATCTGGCGCTCAGGCTATCAGTGATCAGAACGCTCAGGACAAAATGATTGCCGCTCAGACCGCTGCTGGTCGTCGTCAAGCTATGGAAATCATGAGGCAGACGCACATCCAGAATGCTGACCTATCgc                                                                                                                                                                                                   >  1:277630/1‑300 (MQ=255)`  
                                                                                                        `gATACTCTTTGGAATTACTTATGGGTAGGCAATACGTCCCACATTCGTTTCCTCAAGCCTATCGGTGCGGTATTCCATGAAGAGTACACACGAGATGGTCAATTTCAGTTATTTACAATCACGAAAGTAGGATAACCATATGTGTTGGGCAGCCGCAATACCTATCGCTATATCTGGCGCTCAGGCTATCAGTGATCAGAACGCTCAGGCCAAAATGATTGCCGCTCAGACCGCTGCTGGTCGTCGTCAAGCTATGGAAATCATGAGGCAGACGAACATCCAGAATGCTGACCTATCGTTg                                                                                                                                                                                                 <  1:561185/301‑1 (MQ=255)`  
                                                                                                        `aattacgtataggtaggcaatacgtcccacattcgtttcctcaagactatcggtgcggtattccatgaagagtacacacgagatggtcaatttcagttatttacaatcacgaaaggaggataaccatatgtgttgggcagccgcaagacctatcgctatatctggCGCTATATCTGGCGCTCAGGCTATCAGTGGTCAGAACGCTCAGGCCAAAATGATTGCCGCTCAGACCGCTGCTGGTCGTCGTCAAGCTATGGAAATCATGAGGCAGACGAACATCAAGAATGCTGACCTATCGTTg                                                                                                                                                                                                 <  1:342096‑M2/136‑1 (MQ=255)`  
                                                                                                              `ctTTTGAATTACGTATGGGTAGGCAATACGTCCCACATTCGTTTCCTCAAGACTATCGGTGCGGTATTCCATGAAGAGTACACACGAGATGGCCAATCTCAGTTATTTACAATCACGAAAGGAGGATAACCATATGTGTTGGGCAGCTGCAATACCTATCGCTATATTTGGCGCTCAGGCTATCAGTGATCAGAATGCTAAGGCCAAAATGATTGCCGCTCAGACCGCTGCTGGTCGTCGTCAAGCTATGGAAATAACGAGACAGACGAACATCCAGAATGCTGCACTATCGTCGCAAGcc                                                                                                                                                                                           >  1:66731/1‑300 (MQ=255)`  
                                                                                                              `ctTTGGAATTACGTATGGGTAGGCAATACGTCCCACATTCGTTTCCTCAAGACTATCGGTGCGGTATTCCATGAAGAGTACACACGAGATGGTCAATTTCAGTTATTTACAATCACGAAAGGAGGATAACCATATGTGTTGGGCAGCCGCAATACCTATCGCTATATCTGGCGCTCAGGCTATCAGTGATCAGAACGCTCAGGCCAAAATGATTGCCGCTCAGACCGCTGCTGGTCGTCGTCAAGCTATGGAAATCATGAGGCAGACGAACATCCAgctgtctcttatacacatatccgag                                                                                                                                                                                           >  1:101930/1‑277 (MQ=255)`  
                                                                                                                `atgggtaggcaatacgtcccacattcgtttcctcaagactatcggtgcggtattccatgaagagtacacacgagatggtcaatttcagttatttacaatcacgaaaggaggataaccatatgtgttgggcagccgcaatacctatcgctacatctggCGCTATATCTGGCGCTCAGGCTATCAGTGGTCAGAACGCTCAGGCCAAAATGATTGCCGCTCAGACCGCTGCTGGTCGTCGTCAAGCTATGGAAATCATGAGGCAGACGAACATCCAGAATGCTGACCTATCGTTGCAAGCtct                                                                                                                                                                                         >  1:12398‑M2/158‑300 (MQ=255)`  
                                                                                                                        `aCGTATGGGTAGGCAATACGTCCCACATTCGTTTCCTCAAGACTATCGGTGCGGTATTCCATTAAGAGTACACACGAGATGGTCAATTTCAGTTATTTACAATCACGAAAGGAGGATAACCATATGTGTTGGGCAGCCGCAATACCTATCGCTATATCTGGCGCTCAGGCTATCAGTGATCAGAACGCTCAGGCCAAAATGATTGCCGCTCAGACCGCTGCTGGTCGTCGTCAAGCTATGGAAATCATGAGGCAGACGAACATCCAGAATGCTGACCTATCGTTGCACGCTCGAAGTAAAc                                                                                                                                                                                 >  1:316355/1‑301 (MQ=255)`  
                                                                                                                         `caatacgtcccacattcgtttcctcaagactatcggtgcggtattccatgaagagtacacacgagatggtcaatttcagttatttacaatcacgaaaggaggataaccatatgtgttgggcagccgcaatacatatcgctatatctggCGCTATATCTGGCGCTCAGGCTATCAGTGGTCAGAACGCTCAGGCCAAAATGATTGCCGCTCAGACCGCTGCTGGTCGTCGTCAAGCTATGGAAATCATGAGGCAGACGAACATCCAGAATGCTGACATATCGTTGCAAGCTCGAAGTAAACt                                                                                                                                                                                <  1:657286‑M2/153‑1 (MQ=255)`  
                                                                                                                           `tataagagacagaTACGTCCCACATTCGTTTCCTCAAGACTATCGGTGCGGTATTCAATGAAGAGTACACACGAGATGGTCAATTTCAGTTATTTACAATCACGAAAGGAGGATAACCATATGTGATGGGCAGCCGCAATACCTATCGCTATATCTGGCGCTCAGGCTATCAGTGATCAGAACGCTCAGGCCAAAATGATTGCCGCTCAGACCGCTGCTGGTCGTCATCAAGCTATGGAAATCATGAGGCAGACGAACATCCAGAATGCTGACCTATCGTTGCAAGCTCGAAGTAAACTTg                                                                                                                                                                              <  1:774184/289‑1 (MQ=255)`  
                                                                                                                           `atttgtataagagacagTCCCACATTCGTTTCCTCAAGACTATCGGTGCGGTATTCCATGAAGAGTACACACGAGATGGTCAATTTCAGTTATTTACAATCACGAAATGATGATAACCATATGTGTTGGGCAGCCGCAATACCTATCGCTATATCTGGCGCTCAGGCTATCAGTGATCAGAACGCTCAGGCCAAAATGATTGCCGCTCAGACCGCTGCTGGTCGTCGTCAAGCTATGGAAATCATGAGGCATACGAACATCCAGAATGCTGACCTATCGTTGCAAGCTCGAAGTAAACTTg                                                                                                                                                                              <  1:448907/285‑1 (MQ=255)`  
                                                                                                                                  `aGGCAATACGTCCCACATTCGTTTCCTCAAGACTATCGGTGCGGTATTCCATGAAGAGTACACACGAGATGGTCAATTTCAGTTATTTACAATCACGAAAGGAGGATAACCATATGTGTTGGGCAGCCGCAATACCTATCGCTATATCTGGCGCTCAGGCTATCAGTGATCAGAACGCTCAGGCCAAAATGATTGCCGCTCAGACCGCTGCTGGTCGTCGTCAAGCTATGGAAATCATGAGGCAGACGAACATCCAGAATGCTGACCTATCGTTGCAAGCTCGAAGTAAACTTGAGGAAGc                                                                                                                                                                       >  1:303538/1‑301 (MQ=255)`  
                                                                                                                                    `acattcgtttcctcaagactatcggtgcggtattccatgaagagtacacacgagatggtcaatttcagttatttacaatcacgaaaggaggataaccatatgtgttgggcagccgcaatacctatcgctatatctggCGCTATATCTGGCGCTCAGGCTATCAGTGGTCAGAACGCTCAGGCCAAAATGATTGCCGCTCAGACCGCTGCTGGTCGTAGTCAAGCTATGGAAATCATGAGGCGGACGAACATCCAGAATGCTGACCTATCGTTGCAAGCTCGAAGTAAACTTGAGGAAGCGt                                                                                                                                                                     <  1:457158‑M2/164‑1 (MQ=255)`  
                                                                                                                                                  `aTTCGTTTCCTCAAGACTATCGGTGCGGTATTCCATGAAGAGTACACACGAGATGGTCAATTTCAGTTATTTACAATCACGAAAGGAGGATAACCATATGTGTTGGGCAGCCGCAATACCTATCGCTATATCTGGCGCTCAGGCTATCAGTGATCAGAACGCTCAGTCCAAAATTATTGCCGCTCAGACCGCTGCTGGTCGTCGTCAAGCTATGGAAATCATGAGGCAGACGAACATCCAGAATGCTGACCTATCGTTGCAAGCTCGAAGTAAACTTGAggctgtcgcttatacacatctc                                                                                                                                                       >  1:20160/1‑281 (MQ=255)`  
                                                                                                                                                       `tattggtgcgttattccatgaagagtacacacgagatggtcaatttcagttatttacaatcccgaaaggcggataaccatatgtgttgggcagccgcaatacctatcgctatatctggCGCTATATCTGGCGCTCAGGCTATCAGTGGTCAGAACGCTCAGGCCAACATGATTGCCGCTCAGACCGCTGCTGGTCGTCGTCAAGCTATGGAAATCATGAGGCAGTCGAACATCCAGAATGCTGACCTATCGTTGCAAGCTCGAAGTAAACTTGAGGAAGCGTCCGCCGAGTTGACCTcaca                                                                                                                                                  >  1:186805‑M2/119‑301 (MQ=255)`  
                                                                                                                                                        `tcggagatgtgtataagagacagaTTCCATGAAGAGTACACACGAGATGGTCAATTTCAGTTATTTACAATCACGAAAGGAGGATAACCATATGTGTTGGGCAGCCGCAATACCTATCGCTATATCTGGCGCTCAGGCTATCAGTGATCAGAACGCTCAGGCCAAAATGATTGCCGCTCAGACCGCTGCTGGTCGTCGTCAAGCTATGGAAATCATGAGGCAGACGAACATCCAGAATGCTGACCTATCGTTGCAAGCTCGAAGTAAACTTGAGGAAGCGTCCGCCGAGTTGACATCACAg                                                                                                                                                 <  1:350219/278‑1 (MQ=255)`  
                                                                                                                                                          `acaaaagaatataggggggggaatcaaagAAGAGGACACAAGAGAGGGTTAAATTCAGTTATTTACAAACAAGGAAGGAAGGAAAACATATGTGTTGGGCAGCCGCAATACCTATCGCTATATCTGGCGCTCAGGCGATCAGGAATCAGAACGCTCAGGCCAAAATGATTGCCGCTCAGACCGCTGCTGGTCGTCGTCAAGCTATGGAAATCATGAGGCAGACGAACATCCAGAATGCTGACCTATCGTTGCAAGCTCGAAGTAAACTTGAGGAAGCGTCCGCCGAGTTGACCCCACAGaa                                                                                                                                               <  1:284340/273‑1 (MQ=255)`  
                                                                                                                                                           `taagagacagtccatgaagagtacacacgagatggtcaatttcagttatttacaatcacgaaaggaggataaccatatgtgttgggcagccgcaatacctatcgctatatctggCGCTATATCTGGCGCTCAGGCTATCAGTGGTCAGAACGCTCAGGCCAAAATGATTGCCGCTCAGACCGCTGCTGGTCGTCGTCAAGCTATGGAAATCATGAGGCAGACGAACATCCAGAATGCTGACCTATCGTTGCAAGCTCGAAGTAAACTTGAGGAAGCGTCCGCCGAGTTGACCCCACAGAAc                                                                                                                                              <  1:704176‑M2/187‑1 (MQ=255)`  
                                                                                                                                                                `ggtattccatgaagagtacacacgagatggtcaatttcagttatttacaatcacgaaaggaggataaccatatgtgttgggcagccgcaatacctatcgctatatctggCGCTATATCTGGCGCTCAGGCTATCAGTGGTCAGAACGCTCAGGCCAAAATGATTGCCGCTCAGACCGCTGCTGGTCGTCGTCAAGCTATGGAAATCATGAGGCAGACGAACATCCAGAATGCTGACCTATCGTTGCAAGCTCGAAGTAAACTTGAGGAAGCGTCCGCCGAGTTGACGTCACAGAACATGCa                                                                                                                                         >  1:752513‑M2/110‑301 (MQ=255)`  
                                                                                                                                                                 `ggctcggagatgtgtataagagacaggTACATACGAGATGGTCAATTTCAGTTATTTACAATCACGAAAGGAGGATAACCATATGTGTTGGGCAGCCGCAATACCTATCGCTATATCTGGCGCTCAGTCTATCAGTGATCAGAACGCTCAGGCCAAAATGATTGCCGCTCAGACCGCTGCTGGTCGTCGTCAAGCTATGGAAATCATGAGGCAGACGAACATCCAGAATGCTGACCTATCGTTGCAAGCTCGAAGTAAACTTGAGGAAGCGTCCGCCGAGTTGACCCCACAGAACATGCAg                                                                                                                                        <  1:480064/275‑1 (MQ=255)`  
                                                                                                                                                                 `aCTATTGGTGCGGTATTCCATGAAGAGTACACACGAGATGGTCAATTTCAGTTATTTACAATCACGAAAGGAGGATAACCATATGTGTTGGGCAGCCGCAATACCTATCGCTATATCTGGCGCTCAGGCTATCAGTGATCAGAACGCTCAGGCCAAAATGATTGCCGCTCAGACCGCTGCTGGTCGTCGTCAAGCTATGGAAATCATGAGGCAGACGAACATCCAGAATGCTGACCTATCGTTGCAAGCTCGAAGTAAACTTGAGGAAGCGTCCGCCGAGTTGACCACACAGAACATGCAg                                                                                                                                        <  1:361867/301‑1 (MQ=255)`  
                                                                                                                                                                  `gctcggagatgtgtataagagacaggTACGCACGAGATGGTCAATTTCAGTTATTTACAATCACGAAAGGAGGATAACCATATGTGTTGGGCAGCCGCAATACCTATCGCTATATCTGGCGCTCAGGCTATCAGTGATCAGAACGCTCAGGCCAAAATGATTGCCGCTCAGACCGCTGCTGGTCGTCGTCAAGCTATGGAAATCATGAGGCAGACGAACATCAAGAATGCTGACCTATCGTTGCAAGCTCGAAGTAAACTTGAGGAAGCGTCCGCCGAGTTGACCACACAGAACATGCAGa                                                                                                                                       <  1:1306/276‑1 (MQ=255)`  
                                                                                                                                                                      `ccatgaagagtacacaagagatggtcaatttcagttatttacaatcacgaaaggaggataaccatatgtgttgggcagccgcaatacctatcgctatatctggCGCTATATCTGGCGCTCAGGCTATCAGTGATCAGAACGCTCAGGCCAAAATGATTGCCGCTCAGACCGCTGCTGGTCGTCGTCAAGCTATGGAAATCATGAGGCAGACGAACATCCAGAATGCTGACCTATCGTTGCAAGCTCGAAGTAAACTTGAGGAAGCGTCCGCCGAGTTGACCCCACAGAACATGCAGAAGGt                                                                                                                                   >  1:484651‑M2/104‑301 (MQ=255)`  
                                                                                                                                                                      `cGGTGTGGTATTCCATGAAGAGTACACACTAGATGGTCAATTTCAGTTATTTACAATCACGAAAGGAGGATAACCATATGTGTTGGGAAGCCGCAATACGTATCGCTATATCTGGCGCTCAGGCTATCAGTGGTCAGCACGCTCAGGCCAAAATGATTGCCGCTCAGACCGCTGCTGGTCGTCGTCAAGCTATGGAAATCATGAGGCAGACGAACATCCAGAATGCTGACCTATCGTTGCAAGCTCGAAGTAAACTTGAGGAAGCGTACGCCGAGTTGACCTCACAGAACATGCAGAAGGt                                                                                                                                   >  1:336211/1‑301 (MQ=255)`  
                                                                                                                                                                             `ggggctcggagatgtgtataagagacaggTCAATTTCAGTTATTTACAATCACGAAAGTAGGATAACCATATGTGTTGGGCAGCTGCAATACCTATCGCTATATCTGGCGCTCAGGCTATCAGTGATCAGAACGCTCAGGCCAAAATGATTGCCGCTCAGACCGCTGCTGGTCGTCGTCAAGCTATGGAAATCATGAGGCAGATGAACATCCAGAATGCTGACCTATCGTTGCAAGCTCGAAGTAAACTTGAGGAAGCGTACGCCGAGTTGACCCCACAGAACATGCAGAAGGTCCAAGCt                                                                                                                            <  1:418440/274‑1 (MQ=255)`  
                                                                                                                                                                             `gagtacacacgagatggtcaatttcagttatttacaatcacgaaaggaggataaccatatgtgttgggcagccgcaatacctatcgctatatctggCGCTATATCTGGCGCTCAGGCTATCAGTGGTCAGAACGCTCAGGCCAAAATGATTGCCGCTCAGACCGCTGCTGGTCGTCGTCAAGCTATGGAAATCATGAGGCAGACGAACATCCAGAATGCTGACCTATCGTTGCAAGCTCGAAGTAAACTTGAGGAAGCGTCCGCCGAGTTGACCTCACAGAACATGCAGAAGGTCCAAGCt                                                                                                                            <  1:292291‑M2/205‑1 (MQ=255)`  
                                                                                                                                                                               `gtacacatgagatggtcaatttcagttatttacaatcacgaaaggaggataaccatatgtgttgggcagccgcaatacctatcgctatatctggCGCTATATCTGGCGCTCAGGCTATCAGTGGTCAGAACGCTCAGGCCAAAATGATTGCCGCTCAGACCGCTGCTGGTCGTCGTCAAGCTATGGAAATCATGAGGCAGACGAACATCCAGAATGCTGACCTATCGTTGCAAGCTCAAAGTAAACTTGAGGAAGCGTCCGCCAAGTCGACCCCCCAGACCATGCAGAAGGTCAAAGCTAt                                                                                                                          >  1:32483‑M2/95‑301 (MQ=255)`  
                                                                                                                                                                                 `tCCATGAATAGTACACACGAGATGGTCAATTTCAGTTATTTACAATCACGAAAGGAGGATAACCATATGTGTTGGGCAGCCGCAATACCTATCGCTATATATGGCGCTCAGGCTATCAGTGATCAGAACGCTCAGGCCAAAATGATTGCCGCTCAGACCGCTGCTGGTCGTCGTCAAGCTATGGAAATCATGAGGCAGACGAACATCCAGAATGCTGACCTATCGTTGCAAGCTCGAAGTAAACTTGAGGAAGCGTCCGCCGAGTTGACCCCACAGAACATGCAGAAGGTCCAAGCTATTg                                                                                                                        >  1:642301/1‑301 (MQ=255)`  
                                                                                                                                                                                 `tCCATGAAGAGTACACACGAGATGGTCAATTTCAGTTATTTACAATCACGAAATGAGGATAACCATATGTGTTGGGCAGCCGCAATACCTATCGCTATATCTGGCGCTCAGGCTATCAGTGATCAGAACGCTCAGGCCAAAATGATTGCCGCTCAGACCGCTGCTGGTCGTCGTAAAGCTATGGAAAGCATGAGGCATACGAACATACAGAATGCTGACCTATCGTTGCAAGCTCGAAGTAAACTTGAGGAAGCGTACGTCGAGTCGACCTGACAGAACATGCAGAAGGACCAAGCTATTg                                                                                                                        >  1:357175/1‑301 (MQ=255)`  
                                                                                                                                                                                 `tCCATGAAGAGTACACACGAGATGGTCAATTTCAGTTATTTACAATCACGAAAGGATGATAACCATATGTGTTGGGCAGCCGCAATACCTATCGCTATATCTGGCGCTCAGGCTATCAGTGATCAGAACGCTCAGGCCAAAATGATTGCCGCTCAGACCGCTGCTGGTCGTCGTCAAGCTATGGAAATCATGAGGCAGACGAACATCCAGAATGCTGACCTATCGTTGCAAGCTCGAAGTAAACTTGAGGAAGCGTCCGCCGAGTTGACCCCACAGAACATGCAGAATGTCCAAGCTATTg                                                                                                                        >  1:153709/1‑301 (MQ=255)`  
                                                                                                                                                                                  `ccATGAAGAGTACACACGAGATGGTCAATTTCAGTTATTTACAATCACGAAAGGAGGATAACCATATGTGTTGGGCAGCCGCAATACCTATCGCTATATCTGGCGCTCAGGCTATCAGTGATCAGAACGCTCAGGCCAAAATGATTGCCGCTCAGACCGCTGCTGGTCGTCCTCAAGCTATGGAAATCATGAGGCAGACGAACATCAAGAATGCTGACCTATCGTTGCAAGCTCGAAGTAAACTTGAGGAAGCGTCCGCCGAGTTGACCCCACAGAACATGCAGAAGGTACAAGCTATTgg                                                                                                                       >  1:217894/1‑301 (MQ=255)`  
                                                                                                                                                                                    `cacgaaatggtcaatttcagttatttacaatcacgaaaggaggataaccatatgtgttgggcagccgcaatacctatcgctatatctggCGCTATATCTGGCGCTCAGGCTATCAGTGGTCAGAACGCTCAGGCCAAAATGATTGCCGCTCAGCCCGCTGCTGGTCGTCGTCAAGCTATGGAAATCATGAGGCAGACGAACATCCAGAATGCTGACCTATCGTTGCAAGCTCGAAGTAAACTTGAGGAAGCGTCCGCCGAGTTGACCTCACAGAACATGCAGAAGGTCCAAGCTATTGGGt                                                                                                                     <  1:411649‑M2/212‑1 (MQ=255)`  
                                                                                                                                                                                     `agagacagacacacTAGATGGTCAATTTCAGTTATTTACAATCACGAAAGGAGGATAACCATATGTGTTGGGCAGCCGCAATACCTATCGCTATATCTGGCGCTCAGGCTATCAGTGATCAGAACGCTCAGGCCAAAATGATTGCCGCTCAGACCGCTGCTGGTCGTCGTCAAGCTATGGAAATCATGAGGCAGACGAACATCCAGAATGCTGACCTATCGTTGCAAGCTCGAAGTAAACTTGAGGAAGCGTCCGCCGAGTTGACCCAACAGAACATGCAGAAGGTCCAAGCTATTGGGTc                                                                                                                    <  1:521771/293‑1 (MQ=255)`  
                                                                                                                                                                                        `agagTACACACGAGATGGTCAATTTCAGTTATTTACAATTACGAAAGGAGGATAACCATATGTGTTGGGCAGCCGCAATACCTATCGCTATATCTGGCGCTCAGGCTATCAGTGATCAGAACGCTCAGGCCAAAATTATTGCCGCTCAGACCGCTGCTGGTCGTCGTCAAGCTATGGAAATCATGAGGCAGACGAACATCCAGAATGCTGACCTATCGTTGCAAGCTCGAAGTAAACTTGAGGAAGCGTCCGCCGAGTTGACCCCACAGAACATGCAGAAGGTCCAAGCTATTGGGTCTAt                                                                                                                 >  1:36751/1‑301 (MQ=255)`  
                                                                                                                                                                                         `gagTACACACGAGATGGTCAATTTCAGTTATTTACAATCACGAAAGGAGGATAACCATATGTGTTGGGCAGCCGCAATACCTATCGCTATATCTGGCGCTCAGGCTATCAGTGATCAGAACGCTCAGGCCAAAATGATTGCCGCTCAGACCGCTGCTGGTCGTCGTCAAGCTATGGAAATCATGAGGCAGACGAACATCCAGAATGCTGACCTATCGTTGCAAGCTCGAAGTAAACTTGCTGACGCGTCCGCCGAGTTGACCCCACAGAACCTGCCGAAGGTcctgtctcttatcaacatc                                                                                                                >  1:807654/1‑284 (MQ=255)`  
                                                                                                                                                                                           `tggtcaatttcagttatttacaatcacgaaaggaggataaccatatgtggtgggcagcagcaatacctatcgctatatctggCGCTATATCTGGCGCTCAGGCTATCAGTGGTCAGAACGCTCAGGCCAAAATGATTGCCGCTCAGACCGCTGCTGGTCGTCGTCAAGCTATGGAAATCATGAGGTAGACGAACATCCAGAATGCTGACCTATCGTTGCAAGCTCGAAGTAAACTTGAGGAAGCGTCCGCCGAGTTGACCCCACAGAACATGCAGAAGGTCCAAGCTATTTGGTCTATCCg                                                                                                              <  1:444998‑M2/219‑1 (MQ=255)`  
                                                                                                                                                                                           `gTACACACGAGATGGTCAATTTCAGTTATTTACAATCACGAAAGGATGATAACCATATGTGTTGGGCAGCCGCAATACCTATCGCTATATCTGGCGCTCAGGCTATCAGTGATCAGAACGCTCAGGCCAAAATGATTGCCGCTCAGACCGCTGCTGGTCGTCGTCAAGCTATGGAAATCATGAGGCAGACGAACATCCAGAATGCTGACCTATCGTTGCAAGCTCGAAGTAAACTTGAGGAAGCGTCCGCCGAGTTGACCCCACAGAACATGCAGAAGGTCCAAGCTATTGGGTCTATCCg                                                                                                              <  1:727973/301‑1 (MQ=255)`  
                                                                                                                                                                                           `gTACACACGAGATGGTCAATTTCAGTTATTTACAATCACGAAAGGAGGATAACCATATGTGTTGGGCAGCCGCAATACCTATCGCTATATCTGGCGCTCAGGCTATCAGTGATCAGAACGCTCAGGCCAAAATGATTGCCGCTCAGACCGCTGCTGGTCGTCGTCAAGCTATGGAAATCATGAGGCAGACGAACATCCAGAATGCTGACCTATCGTTGCAAGCTCGAAGTAAACTTGAGGAAGCGTCCGCCGAGTTGACCCAACAGAACATGCAGAAGGTCCAAGCTATTGGGTCTATCCg                                                                                                              >  1:749143/1‑301 (MQ=255)`  
                                                                                                                                                                                             `ccacacGAGATGGTCAATTTCAGTTATTTACAATCACGAAAGGAGGATAACCATATGTGTTGGGCAGCCGCAATACCTATCGCTATATCTGGCGCTCAGGCTATCAGTGATCAGAACGCTCAGGCCAAAATGATTGCCGCTCAGACCGCTGCTGGTCGTCGTCAAGCTATGGAAATCATGAGGCAGACGAACATCCAGAATGCTGACCTATCGTTGCAAGCTCGAAGTAAACTTGAGGAAGCGTCCGCCGAGTTGACCCCACAGAACATGCAGAAGGTCCAAGCTATTGGGTCTATCCGAg                                                                                                            >  1:267855/2‑301 (MQ=255)`  
                                                                                                                                                                                                       `gttatttacaatcacgaaaggaggataaccatatgtgttgggcagccgcaatacctatcgctatatctggCGCTATATCTGGCGCTCAGGATATCAGTGGTCAGAACGCTCAGGCCAAAATGATTGCCGCTCAGACCGCTGCTGGTCGTCGTCAAGCTATGGAAATCATGAGGCAGACGAACATCCAGAATGCTGACCTATCGTTGAAAGCTCGAAGTAAACTTGAGGAAGCGTCCGCCGAGTTGACCCCACAGAACATGCAGAAGGTCCAAGCTATTGGGTCTATCCGAGCGGCTATCgg                                                                                                  <  1:616607‑M2/231‑1 (MQ=255)`  
                                                                                                                                                                                                        `ggTCAATTTCAGTTATTTACAATCACGAAAGGAGGATAACCATATGTGTTGGGCAGCCGCAATACCTATCGCTATATCTGGCGCTCAGGCTATCAGTGATCAGAACGCTCAGGCCAAAATGATTGCCGCTCAGACCGCTGCTGGTCGTCGTCAAGCTATGGAAATCATGAGGCAGACGAACATCCAGAATGCTGACCTATCGTTGCAAGCTCGAAGTAAACTTGAGGAAGCGTCCGCCGAGTTGACCCCACAGAACATGCAGAAGGTCCAAGCTATTGGGTCTATCCGAGCGGCTATCGga                                                                                                 >  1:231797/1‑301 (MQ=255)`  
                                                                                                                                                                                                         `taagagacagcacgaaaggaggataaccatatgtgttgggcagccgcaatacctatcgctatatctggCGCTATATCTGGCGCTCAGGCTATCAGTGATCAGAACGCTCAGGCCAAAATGATTGCCGCTCAGACCGCTGCTGGTCGTCGTCAAGCTATGGAAATCATGAGGCAGACGAACATCCAGAATGCTGACCTATCGTTGCAAGCTCGAAGTAAACTTGAGGAAGCGTCCGCCGAGTTGACCCCACAGAACATGCAGAAGGTCCAAGCTATTGGGTCTATCCGAGCGGCTATAGgag                                                                                                <  1:16540‑M2/233‑1 (MQ=255)`  
                                                                                                                                                                                                         `gTCAATTTCAGTTATTTACAATCACGAAAGGAGGATAACCATATGTGTTTGGCAGCCGCAATACCTATCGCTATATCTGGCGCTCAGGCTATCAGTGATCAGAACGCTCAGGCCAAAATGATTGCCGCTCAGACCGCTGCTGGTCGTCGTCAAGCTATGGAAATCATGAGGCAGACGAACATCCAGAATGCTGACCTATCGTTGCAAGCTCGAAGTAAACTTGAGGAAGCGTCAGCCGAGTTGACCCCACAGAACATGCAGAAGGTCCAAGCTATTGGGTCTATCCGAGCGGCTATCGgag                                                                                                >  1:716087/1‑301 (MQ=255)`  
                                                                                                                                                                                                         `gTCAATTTCAGTTATTTACAATCACGAAAGGAGGATAACCATATGTGTTGGGCAGCCGCAATACCTAGCGCTATATCTGGCGCTCAGGCTATCAGTGATCAGAACGCTAAGGCCAAAATGATTGCCGATCAGACCGCTGCTGGTCGTCGTCAAGCTATGGAAATCATGAGGCAGACGAACATCCAGAATGCTGACCTATCGTTGCAAGCTCGAAGTAAACTTGAGGAAGCGTCCACCGAGTTGACCCCACAGAACATGCAGAAGGTCCAAGCTATTGGGTCTATCCGAGCGGCTATCGgag                                                                                                <  1:86590/301‑1 (MQ=255)`  
                                                                                                                                                                                                         `aaggagatgtgtataagaaacagtaaccagatgtgttgggcagccgcaatacctatcgctatatctgtCGCTATATCTGGCGCTCAGGCTATCAGTGGTCATAACGCTCAGGCCAAAATGATTGCCGCTCAGACCGCTGCTGGTCGTCGTCAAGCTATGGAAATCATGAGGCAGACGAACATCCAGAATGCTGACCTATCGTTGCAAGCTCTAAGTAAACTTGAGGAAGCGTCCGCCGAGTTGACCTCACAGAACATGCAGAAGGTCCAAGCTATTGGGTCTATCCGAGCGGCTATCGgag                                                                                                <  1:264062‑M2/233‑1 (MQ=255)`  
                                                                                                                                                                                                             `ggctcggagatgtgtataagagacaggaggATAACCATATGTGTTGGGCAGCCGCAATACCTATCGCTATATCTGGCGCTCAGGCTATCAGTGATAAGAACGCTCAGGCCAAAATGATTGCCGCTCAGACTGCTGCTGGTCGTCGTCAAGCTATGGAAATCATGAGGCAGACGAACATCCAGAATGCTGACCTATCGTTGCAAGCTCGAAGTAAACTTGAGGAAGCGTCAGCCGAGTTGACCCCACAGAACATGCAGAAGGTCCAAGCTATTGGGTCTATCCGAGCGGCTATCGGagagag                                                                                            <  1:402729/277‑1 (MQ=255)`  
                                                                                                                                                                                                             `aTTTCAGTTATTTACAATCACGAAAGGAGGATAACCATATGTGTTGGGCAGCCGCAATACCTATAGCTATATCTGGAGCTCAGGATATCAGTGATCAGAACGCTCAGGCCAAAATGATTGCCGCTCAGACCGCTGCTGGTCGTCGTCAAGCTATGGAAATCATGAGGCAGACGAACATCCAGAATGCTGACCTATCGTTGCAAGCTCGAAGTAAACTTGAGGAAGCGTCCGCCGAGTTGACCCCACAGAACATGCAGAAGGTCCAAGCTATTGGGTCTATCCGAGCGGCTATCGGagagag                                                                                            >  1:621973/1‑301 (MQ=255)`  
                                                                                                                                                                                                                   `tgggctaggagatgggtataagagacagccATATGTGTTGGGCAGCCGCAATACCTATCGCTATATCTGGCGCTCAGGCTATCAGTGATCAGAACGCTCAGGCCAAAATGATTGCCGCTCAGACCGCTGCTGGTCGTCGTCAAGCTATGGAAATCATGAGGCAGACGAACATCCAGAATGCTGACCTATCGTTGCAAGCTCGAAGTAAACTTGAGGAAGCGTCCGCCGAGTTGACCCCACAGAACATGCAGAAGGTCCAAGCTATTGGGTCTATCCGAGCGGCTATCGGAGAGAGTATGCt                                                                                      <  1:517339/273‑1 (MQ=255)`  
                                                                                                                                                                                                                   `taagagacagaTCATGAAAGGAGGATAACCATATGTGTTGGGCAGCCGCAATACCTATCGCTATATCTGGCGCTCAGGCTATCAGTGATCAGAACGCTCAGGCCAAAATGATTGCCGCTCAGACCGTTGCTGGTCGTCGTCAAGCTATGGAAATCATGAGGCAGACGAACATCCAGAATGCTGACCTATCGTTGCAAGCTCGAAGTAAACTTGAGGAAGCGTCCGCCGAGTTGACCCCACAGAACATGCAGAAGGTCCAAGCTATTGGGTATATCCGAGCGGCTATCGGAGAGAGTATGCt                                                                                      <  1:572178/291‑1 (MQ=255)`  
                                                                                                                                                                                                                   `gggctcggagatgtgtataagagacagaCCATATGTGTTGGGCAGCCGCAATACCTATCGCTATATCTGGCGCTCAGGCTATCAGTGATCAGAACGCTCAGGCCAAAATGATTGCCGCTCAGACCGCTGCTGGTCGTCGTCAAGCTATGGAAATCATGAGGCAGACGAACATCCATAATGCTGACCTATCGTTGCAAGCTCGAAGTAAACTTGAGGAAGCGTCCGCCGAGTTGACCCCACAGAACATGCAGAAGGTCCAAGCTATTGGGTCTATCCGAGCGGCTATCTGAGAGAGTATGCt                                                                                      <  1:319650/274‑1 (MQ=255)`  
                                                                                                                                                                                                                   `gTTACTTACAATCACGAAAGGAGGATAACCATATGTGTTGGGCAGCCGCAATACCTATCGCTATATCTGGCGCTCACGCTATCAGTGATCAGAACGCTCAGGCCAAAATGATTGCCGCTCAGACCGCTGCTGGTCGTCGTCAAGCTATGGAAATCATGAGGCAGACGAACCTCCAGAATGCTGAACTATCGTTGCAAGCTCGAAGTAAACTTGAGGAAGAATCCGCCGAGTTGACCCCACAGAACATGCAGAAGGTCCAAGCTATTGGGTCTATCTGAGCGGCTATCGGAGAGAGTATGCt                                                                                      <  1:556591/301‑1 (MQ=255)`  
                                                                                                                                                                                                                   `catgaaaggaggataaacatatgtgttgggcagccgcaatacctatcgctatatctggCGCTATATCTGGCGCTCAGGCTATCAGTGGTCAGAACGCTCAGGCCAAAATGATTGCCGCTAAGACCGATGCTGGTCGTCGTCAAGCTATGGAAATCATGAGGCAGACGAACATCCAGAATGCTGACCTATCGTTGCAAGCTCGAAGTAAACTTGAGGAAGCGTCCGCCGAGTTGACCACACAGAACATGCAGAAGGTCCAAGCTATTGGGTCTATCCGAGCGGCTATCGGAGAGAGTATGCt                                                                                      <  1:25825‑M2/243‑1 (MQ=255)`  
                                                                                                                                                                                                                   `agatgtgtataagagacagggaggaTAACCATATGTGTTGGGCAGCCGCAATACCTATCGCTATATCTGGCGCTCAGGCTATCAGTGATCAGAACGCTCAGGCCAAAATGATTGCCGCTCAGACCGCTGCTGGTCGTCGTCAAGCTATGGAAATCATGAGGCAGACGAACATCCAGAATGCTGACCTATCGTTGCAAGCTCGAAGTAAACTTGAGGAAGCGTCCGCCGAGTTGACCCCACAGAACATGCAGAAGGTCCAAGCTATTGGGTCTATCCGAGCGGCTATCGGAGAGAGTATGCt                                                                                      <  1:329585/282‑1 (MQ=255)`  
                                                                                                                                                                                                                          `ggctcggagatgtgtataagagacagtgtgTTGGGCAGCCGCAATACCTATCGCTATATCTGGCGCTCAGGCTATCAGTGATCAGAACGCTCAGGCCAAAATGATTGCCGCTCAGACCGCTGCTGGTCGTCGTCAAGCTATGGAAATCATGAGGCAGACGAACATCCAGAATGCTGACCTATCGTTGCAAGCTCGAAGTAAACTTGAGGAAGCGTCCGCCGAGTTGACCCCACAGAACATGCAGAAGGTCCAAGCTATTGGGTCTATCCGAGCGGCTATAGGAGAGAGTATGCTTGAAGGt                                                                               <  1:68209/275‑1 (MQ=255)`  
                                                                                                                                                                                                                          `aCAATCACGAAAGGAGGATAACCATATGTGTTGGGCAGCCGCAATACCTATCGCTATATCTGGCGCTCAGGCTATCAGTGATCAGAACGCTAAGGCCAAAATGATTGCCGCTCAGACCGCTGCTGGTCGTCGTCAAGCTATGGAAATCATGAGGCAGACGAACATCCAGAATGCTGACCTATCGTTGCAAGCTCGAAGTAAACTTGAGGAAGCGTCCGCCGAGTTGACCCCACAGAACATGCAGAAGGTCCAAGCTATTGGGTCTATCCGAGCGGCTATCGGAGAGAGTATGCTTGAAGGt                                                                               <  1:755803/301‑1 (MQ=255)`  
                                                                                                                                                                                                                               `cACGAAAGGAGGATAACCATATGTGTTGGGCAGCCGCAATACCTATCGCTATATCTGGCGCTCAGGCTATCAGTGATCAGAACGCTCAGGCCAAAATGATTGCCGCTCAGACCGCTGCTGGTCGTCGTCAAGCTATGGAAATCATGAGGCAGACGAACATCCAGAATGCTGACCTATCGTTGCAAGCTCGAAGTAAACTTGAGGAAGCGTCCGCCGAGTTGACCCCACAGAACATGCAGAAGGTCCAAGCTATTGGGTCTATCCGAGCGGCTATCGGAGAGCGTATGCTTGAAggcagtcc                                                                          >  1:471045/1‑295 (MQ=255)`  
                                                                                                                                                                                                                               `cACGAAAGGAGGATAACCATATGTGTTGGGCAGCAGCAATACCTATCGCTATATCTGGCGCTCAGGCTATCAGTGATCAGAACGCTCAGGCCAAAATGATTGCCGCTCAGACCGCTGCTGGTCGTCGTCAAGCTATGGAAATCATGAGGCAGACGAACATCCAGAATGCTGACCTATCGTTGCAAGCTCGAAGTAAACTTGAGGAAGCGTCCACCGAGTTGACCCCACAGAACATGCAGAAGGTCCAAGCTATTGGGTCTATCCGAGCGGCTATCGGAGAGAGTATGCTTGAAGGTTCCTc                                                                          >  1:310713/1‑301 (MQ=255)`  
                                                                                                                                                                                                                                 `tataagagacagaaCCATATGTGTTGGGCAGCCGCAATACCTATCGCTATATCTGGCGCTCAGGCTATCAGTGATCAGAACGCTCAGGCCAAAATGATTGCCGCTCAGACCGCTGCTGGTCGTCGTCAAGCTATGGAAATCATGAGGCAGACGAACATCCAGAATGCTGACCTATCGTTGCAAGCTCGAAGTAAACTTGAAGAAGCGTCCGCCGAGTTGACACCACAGAACATGCAGAAGGTCCAAGCTATTGGGTCTATCCGAGCGGCTATCGGAGAGAGTATGCTTGAAGGTTCATCaa                                                                        <  1:162680/289‑1 (MQ=255)`  
                                                                                                                                                                                                                                   `ccatatgtgttgggcagccgcaatacctatcgctatatctggCGCTATATCTGTCGCTCAGGCTATCAGTGGTCAGAACGCTCAGGCCAAAATGATTGCCGCTCAGACCGCTGCTGGTCGTCGTCAAGCTATGGAAATCATGAGGCAGACGAACATCCAGAATGCTGACCTATCGTTGCAAGCTCGAAGTAAACTTGAGGAAGCGTCCGCCGAGTTGACCCCACAGAACATGCAGAAGGTCCAAGCTATTGGGTCTATCCGAGCGGCTatctgagagagtatgctctgtctcttatacaca                                                                      >  1:253312‑M2/43‑285 (MQ=255)`  
                                                                                                                                                                                                                                    `cagaagtgttgggcagccgcaatacctatcgctatatctggCGCCATATCTGGCGCTCAGGCTATCAGTGGTCAGAACGCTCAGGCCAAAATGATTGCCGCTCAGACCGCTGCTGGTCGTCGTCAAGCTATGGAAATCATGAGGCAGACGAACATCCAGAATGCTGACCTATCGTTGCAAGCTCGAAGTAAACTTGAGGAAGCGTCCGCCGAGTTGACCTCACAGAACATGCAGAAGGTCCAAGCTATTGGGTCTAACCGAGCGGCTATCGGAGAGAGTATGCTTGAAGGTTCCTCAATgg                                                                     <  1:605593‑M2/260‑1 (MQ=255)`  
                                                                                                                                                                                                                                             `aaCCATATGTGTTGGGCAGCCGCAATACCTATCGCTATATCTGGCGCTCAGGCTATCAGTGATCAGAACGCTCAGGCCAAAATGATTGCCGCTCAGACCGCTGCTGGTCGTCGTCAAGCTATGGAAATCATGAGGCAGACGAACATCCAGAATGCTGACCTATCGTTGCAAGCTCGAAGTAAACTTGAGGAAGCGTCCGCCGAGTTGAACCCACAGAACATGCAGAAGGTCCAAGCTATTGGGTCTATCCGAGCGGCTATCGGAGAGAGTATGCtctgtctcttatacacatctccgagcc                                                            >  1:819895/1‑275 (MQ=255)`  
                                                                                                                                                                                                                                              `aCCATATGTGTTGGGCAGCCGCAATACCTATCGCTATATCTGGCGCTCAGGCTATCAGTGATCAGAACGCTCAGGCCAAAATGATTGCCGCTCAGACCGCTGCTGGTCGTCGTCAATCTATGGAAATCATGAGGCAGACGAACATCCAGAATGCTGACCTATCGTTGCAAGCTCGAAGTAAACTTGAGGAAGCGTCCGCCGAGTTGACCCCACAGAACATGCAGAAGGTCCAAGCTATTGGGTCTATCCGAGCGGCTATCGGAGAGAGTATGCTTGAAGGTTCCTCAATggctgtctcttt                                                           >  1:452989/1‑291 (MQ=255)`  
                                                                                                                                                                                                                                                 `atatGTGTTGGGCAGCCGCAATACCTATCGCTATATCTGGCGCTCAGGCTATCAGTGATCAGAACGCTCAGGCCAAAATGATTGCCGCTCAGACCGCTGCTGGTCGTCGTCAAGCTATGGAAATCATGAGGCAGACGAACATCCAGAATGCTGACCTATCGTTGCAAGCTCGAAGTAAACTTGAGGAAGCGTCAGCCGAGTTGACCCCACAGAACATGCAGAAGGTCCAAGCTATTGGGTCTATCCGAGCGGCTATCGGAGAGAGTATGCTTGAAGGTTCCTCAATGGACCGCATTAAGCg                                                        <  1:23985/301‑1 (MQ=255)`  
                                                                                                                                                                                                                                                  `tatGTGTTGGGCAGCCGCAATACCTATCGCTATATCTGGCGCTCAGGCTATCAGTGATCAGAACGCTCAGGCCAAAATGATTGCCGCTCAGACCGCTGCTGGTCGTCGTCAAGCTATGGAAATCATGAGGCAGACGAACATCCAGAATGCTGACCTATCGTTGCAAGCTCGAAGTAAACTTGAGGAAGCGTCCGCCGAGTTGACCCCACAGAACATGCAGAAGGTCCAAGCTATTGGGTCTATCCGAGCGGCTATCGGAGAGAGTATGCTTGAAGGTTCCTCAAtctgtctcttatacaca                                                       >  1:429156/1‑285 (MQ=255)`  
                                                                                                                                                                                                                                                    `ccgcaatacctatcgctatatctggCGCTATATCTGGCGCTCAGGCTATCAGTGGTCAGAACGCTCAGGCCAAAATGATTGCCGCTCAGACCGCTGCTGGTCGTCGTCAAGCTATGGAAATCATGAGGCAGACGAACATCCAGAATGCTGACCTATCGTTGCAAGCTCGAAGTAAACTTGAGGAAGCGTCCGCCGAGTTGACCTCACAGAACATGCAGAAGGTCCAAGCTATTGGGTCTATCCGAGCGGCTATCGGAGAGAGTATGCTTGAAGGTTCCTCAATGGACCGCATTAAGCGagg                                                     >  1:424474‑M2/26‑300 (MQ=255)`  
                                                                                                                                                                                                                                                         `atacctatcgctatatctggCGCTATATCTGGCGCTCAGGCTATCAGTGGTCAGAATGCTCAGGCCAAAATGATTGCCGCTCAGACCGCTGCTGGTCGTCGTCAAGCTATGGAAATCATGAGGCAGACGAACATCCAGAATGCTGACCTATCGTTGCAAGCTCGAAGTAAACTTGAGGAAGCGCCCGCCGAGTTGACCACACAGAACATGCAGAAGGTCCAAGCTATTGGGTGTATCCGAGCGGCTATCGGATAGAGTATGCTTGAAGGTTCATCAATGGAGCGCATTAAGCGAGTCACAg                                                >  1:246‑M2/21‑301 (MQ=255)`  
                                                                                                                                                                                                                                                           `gacAGCCGCAATACCTATCCCTATATCTGGCGCTCAGGCTATCAGTGATCAGAACGCTCAGGCCAAAATGATTGCCGCTCAGACCGCTGCTGGTCGTCGTCAAGCTATGGAAATCATGAGGCAGACGAACATCCAGAATGCTGACCTATCGTTGCAAGCTCGAAGTAAACTTGAGGAAGTGTCCGCCGAGTTGACCCAACAGAACATGCAGAAGGTCCAAGCTATTGGGTCTATCCGAGCGGCTATCGGAGAGAGTATGCTTGAAGGTTCCTCCATGGACCGCATTAAGcctgtctcttat                                              >  1:173604/3‑290 (MQ=255)`  
                                                                                                                                                                                                                                                           `accaatcgctatatctggCGCTATATCTGGCGCTCAGGCTATCAGTGGTCAGAACGCTCAGGCCAAAATGATTGCCGCTCATACCGCTGCTGGTCGTCGTCAAGCTATGGAAATCATGAGGCAGACGAACATCCAGAATGCTGACCTATCGTTGCAAGCTCGAAGTAAACTTGAGGAAGCGTCCGCCGAGTTGACCTCACAGAACATGCAGAAGGTCCAAGCTATTGGGTCTATCCGAGCGGCTATCGGAGAGAGTATGcttgaaggttcctcaatggacctgtctcttatacacatctcc                                              >  1:602243‑M2/19‑281 (MQ=255)`  
                                                                                                                                                                                                                                                            `cctattgctatatctggCGCTATATCTGGCGCTCAGGCTATCAGTGGTCAGAACGCTCAGGCCAAAATGATTGCCGCTCAGACCGCTGCTGGTCGTCGTCAAGCTATGGAAATCATGAGGCAGACGAACATCCAGAATGCTGACCTATCGTTGCAAGCTCGAAGTAAACTTGAGGAAGCGTCCGCCGAGTTGACCTCACAGAACATGCAGAAGGTCCAAGCTATTGGGTCTATCCGAGCGGCTATCGGAGAGAGTATgcttgaaggttcctcaatggacctgtctcttatacacatctccc                                             >  1:77944‑M2/18‑280 (MQ=255)`  
                                                                                                                                                                                                                                                               `gCCGCAATACCTATCGCTATATCTGGCGCTCAGGCTATCAGTGGTCAGAACGCTCAGGCCAAAATGATTGCCGCTAAGACCGCTGCTGGTCGTCGTCAAGCTATGGAAATCATGAGGCAGACGAACATCCAGAATGCTGACCTATCGTTGCAAGCTCGAAGTAAACTTGAGGAAGCGTCCGCCGAGTTGACCTCACAGAACATGCAGAAGGTACAAGCTATTGGGTCTATCCGAGCGGCTATCGGAGAGAGTATGCTTGAAGGTTCCTCAATGGACCGCATTAAGCGAGGCACAGAAGGAc                                          >  1:439780/1‑301 (MQ=255)`  
                                                                                                                                                                                                                                                               `gCCGCAATACCTATCGCTATATCTGGCGCTCAGGCTATCAGTGATCAGAACGCTCAGGCCAAAATGATTGCCGCTCAGACCGCTGCTGGTCGTCGTCAAGCTATGGAAATCATGATGCAGACGAACATCCAGAATGCTGACCTATCGTTGCAAGCTCGAAGTAAACTTGAGGAAGCGTCCGCCGAGTTGACCCCACAGAACATGCAGAAGGTCCAAGCTATTGGGTCTATCCGAGCGGCTATCGGAGAGAGTATGCTTGAAGGTTCCTCAATGGACCGCATTAAGCGAGTCACAGAAGGAc                                          <  1:194456/301‑1 (MQ=255)`  
                                                                                                                                                                                                                                                               `gCCGCAATACCTATCGCTATATCTGGCGCTCAGGCTATCAGTGATCAGAACGCTCAGGCCAAAATGATTGCCGCTCAGACCGCTGCTGGTCGTCGTCAAGCTATGGAAATCATGAGGCAGACGAACATCCAGAATGCTGACCTATCGTTGCAAGCTCGAAGTAAACTTGAGGAAGCGTCCGCCGAGTTGACCCCACAGAACATGCAGAAGGTCCAAGCTATTGGGTCTATCCGAGCGGCTATCGGAGAGAGTATGCTTGAAGGTTCCTCAATGGACCGCATTAAGCGAGTCACAGAAGGAc                                          <  1:595449/301‑1 (MQ=255)`  
                                                                                                                                                                                                                                                               `gCCGCAATACCTATCGCTATATCTGGCGCTCAGGCTATCAGTGATCAGAACGCTCAGGCCAAAATGATTGCCGCTAAGACCGCTGCTGGTCGTCGTCAAGCTATGGAAATCATGAGGCAGACGAACATCCAGAATGCTGACCTATCGTTGCAAGCTCGAAGTAAACTTGAGGAAGCGTCCGCCGAGTTGACCCCACAGAACATGCAGAAGGTCCAAGCTATTGGGTCTATCCGAGCGGCTATCGGAGAGAGTATGCTTGAAGGTTCCTCAATGGACCGCATTAAGCGAGTCACAGAAGGAc                                          >  1:693318/1‑301 (MQ=255)`  
                                                                                                                                                                                                                                                                  `gctatatctggCGCTATATCTGGCGCTCAGGCTATCAGTGGTCAGAACGCTCAGGACAAAATGATTGCCGCTCAGACCGCTGCTGGTCGTCGTCAAGCTATGGAAATCATGAGGCAGACGAACATCCAGAATGCTGACCTATCGTTGCAAGCTCGAAGTAAACTTGAGGAAGCGTCCGCCGAGTTGACCCCACAGAACATGCAGAAGGTCCAAGCTATTGGGTCTATCCGAGCGGCTATCGGAGAGAGTATGCTTGAAGGTTCCTCAATGGACAgcattaagcgagtcctgtctcttatac                                       >  1:649465‑M2/12‑288 (MQ=255)`  
                                                                                                                                                                                                                                                                     `agagaaagCGCTATATCTGGCGCTCAGGCTATCAGTGATCATAACGCTCAGGCCAAAATGATTGCCGCTCAGACCGCTGCTGGTCATCGTCAAGCTATGGAAATCATGAGGCAGACGAACATCCAGAATGCTGACCTATCGTTGCAAGCTCGAAGTAAACTTGAGGAAGCGTCCGCCGAGTTGACCCCACAGAACATGCAGAAGGTCCAAGCTATTGTGTCTATCCGAGCGGCTATCGGAGAGAGTATGCTTTAAGGTTCCTCAATGGACCGCATTAAGCGAGTCACAGAAGGACAGTTCa                                    <  1:500263‑M2/293‑1 (MQ=255)`  
                                                                                                                                                                                                                                                                       `atgtgtataagagacaggCGCTCAGGCTATCAGTGGTCAGAACGCTCAGGCCAAAATGATTGCCGCTCAGACCGCTGCTGGTCGTCGTCAAGCTATGGAAATCATGAGGCAGACGAACATCCAGAATGCTGACCTATCGTTGCAAGCTCGAAGTAAACTTGAGGAAGCGTCCGCCGAGTTGACCACACAGAACATGCAGAAGGTCCAAGCTATTGGGTCTATCCGAGCGGCTATCGGAGAGAGTATGCTTGAAGGTTCCTCAATGGACCGCATTAAGCGAGTCACAGAAGGACAGTTCAtt                                  <  1:791680/285‑1 (MQ=255)`  
                                                                                                                                                                                                                                                                           `ggCGCTATATCTGGCGCTCAGGCTATCAGTGGTCAGAACGCTCAGGCCAAAATGATTGCCGCTCAGACCGCTGCTGGTCGTCGTCAAGCTATGGAAATCATGAGGCAGACGAACATCCAGAATGCTGACCTATCGTTGCAAGCTCGAAGTAAACTTGAGGAAGCGTCCGCCGAGTTGACCCCACAGAACATGCAGAAGGTCCAAGCTATTGGGTCTATCCGATCGGCTATCGGAGAGAGTATGCTTGAAGGTTCCTCAATGGACCGCATTAAGCGAGTCACAGAAGGACAGTTCATTCggg                              <  1:393156‑M2/299‑1 (MQ=255)`  
                                                                                                                                                                                                                                                                           `ggCGCTATATCTGGCGCTCAGGCTATCAGTGGTCAGAACGCTCAGGCCAAAATGATTGCCGCTCAGACCGCTGCTGGTCGTCGTCAAGCTATGGAAATCATGAGGCAGACGAACATCCAGAATGCTGACCTATCGTTGCAAGCTCGAAGTAAACTTGAGGAAGCGTCCGCCGAGTTGACCCCACAGAACATGCAGAAGGTCCAAGCTATTGGGTCTATCCGAGCGGCTATCGGAGAGAGTATGCTTTAAGGTTCCTCAATGGACCGCATTAAGCGAGTCACAGAAGGACAGTTCCTTCtgg                              >  1:379742‑M2/3‑301 (MQ=255)`  
                                                                                                                                                                                                                                                                            `gCGCTATATCTGGCGCTCAGGCTATCAGTGGTCAGAATGCTCAGGCCAAAATGATTGCCGCTCAGACCGCTGCTGGTCGTCGTCAAGCTATGGAAATCATGAGGCAGACGAACATCCAGAATGCTGACCTATCGTTGCAAGCTCGAAGTAAACTTGAGGAAGCGTCCGCCGAGTTGACCTCACAGAACATGCAGAAGGTCCAAGCTATTGGGTCTATCCGAGCGGCTATCGGAGAGAGTATGCTTGAAGGTTCCTCTATGGACCGCATTAAGCGAGTCACAGAAGGCCAGTTCATTCTGGa                             >  1:700595‑M2/2‑301 (MQ=255)`  
                                                                                                                                                                                                                                                                                   `atCTGGCTCTCAGGCTATCAGTGATCAGAACGCTCAGGCCAAAATGATTGCCGCTCAGACCGCTGCTGGTCGTCGTCAAGCTATGGAAATCATGAGGCAGACGAACATCCAGAATGCTGACCTATCGTTGCAAGCTCGAAGTAAACTTGAGGAAGCGTCCGCCGAGTTGACCCCACAGAACATGCAGAAGGTCCAAGCTATTGGGTCTATCCGAGCGGCTATCGGAGAGAGTAGGCTTGAAGGTTCCTCAATGGACGGCATTAAGCGAGTCACAGAAGGACAgctgtctcttatacacatt                      >  1:488549/1‑283 (MQ=255)`  
                                                                                                                                                                                                                                                                                       `ggCGCTCAGGCTATCAGTGATCAGAACGCTGAGGCCAAAATGATAGCCGCTCAGACCGCTGCTGGTCGTCGTCAAGCTATGGAAATCATGAGGCAGACGAACATCCAGAAGGCGGACCTATCGTTGCAAGCTCGAAGTAAACTTGAGGAAGCGTCCGACAAGTTGACCCAACAGAACATGCAGAAGGTCCAAGCTATTGGGTCTATCCGAGCGGCTATCGGAGAGAGTATGCTTGAAGGTTCCTCAATGGACCGAATTAAGCGAGTCACAGAAGGACAGTTCATTCGGGAAGCAAATATgg                  <  1:677548/301‑1 (MQ=255)`  
                                                                                                                                                                                                                                                                                               `ggCTATCAGTGATCAGAACGCTCAGGCCAAAATGATTGCCGCTCTGACCGCTGCTGGTCGTCGTCAAGCTATGGAAATCATGAGGCAGACGAACATCCAGAATGCTGACCTATCGTTGCAAGCTCGAAGTAAACTTGAGGAAGCGTCAGCCGAGTTGACCCCACAGAACATGCAGAAGGTCCAAGCTATTGGGTCTATCCGAGCGGCTATCGGAGAGAGTATGCTTGAAGGTTCCTCAATGGACAGCATTAAGCGAGTCACAGAAGGACAGTTCATTcctgtctcttatacacatctccgg          >  1:510252/1‑278 (MQ=255)`  
                                                                                                                                                                                                                                                                                                   `aTCAGTGATCAGAACGCTCAGGCCAAAATGATTGCCGCTCAGACCGCTGCTGGTCGTCGTCAAGCTATGGAAATCATGAGGCAGACGAACATCCAGAATGCTGACCTATCGTTGCAAGCTCGAAGTAAACTTGAGGAAGCGTCCGCCGAGTTGACCCCACAGAACATGCAGAAGGTCCAAGCTATTGGGTCTATCCGAGCGGCTATCGGAGAGAGTATGCTTGAAGGTTCCTCAATGGACCGCATTAAGCGAGTCACAGAAGGACAGTTCATTCGGGAAGCCAATATGGTAACTGAGAACt      <  1:345286/301‑1 (MQ=255)`  
                                                                                                                                                                                                                                                                                                       `gTGATCAGAACGCTCAGGCCAAAATGATTGCCGCTCAGACCGCTGCTGGTCGTCGTCAAGCTATGGAAATCATGAGACAGACGAACATCCAGAATGCTGACCTATCGTTGCAAGCTCGAAGTAAACTTGAGGAAGCGTCCGCCGAGTTGACCCCACAGAACATGCAGAAGGTCCAAGCTATTGGGTCTATCCGAGCGGCTATCGGAGAGAGTATGCTTGAAGGTTCCTCAATGGACCGCATTAAGCGAGTCACAGAAGGACAGTTCATTCTGGAAGCCAATATGGTAAcctgtctcttata  >  1:18914/1‑289 (MQ=255)`  

                                                                                                                                                                                                                                                                                                          `|`                                                                                                                                                                                                                                                                                                           
`GCTGGTTCGTTACGAGCGACCAAGTGTGGCGACTTAGTGGAAAGGCTAAGCGAAAGTTCCGTAAGTTAATCATGGAGTATCGCGATAAGATGCTTGAGAAGTATGATACTCTTTGGAATTACGTATGGGTAGGCAATACGTCCCACATTCGTTTCCTCAAGACTATCGGTGCGGTATTCCATGAAGAGTACACACGAGATGGTCAATTTCAGTTATTTACAATCACGAAAGGAGGATAACCATATGTGTTGGGCAGCCGCAATACCTATCGCTATATCTGGCGCTCAGGCTATCAGTGGTCAGAACGCTCAGGCCAAAATGATTGCCGCTCAGACCGCTGCTGGTCGTCGTCAAGCTATGGAAATCATGAGGCAGACGAACATCCAGAATGCTGACCTATCGTTGCAAGCTCGAAGTAAACTTGAGGAAGCGTCCGCCGAGTTGACCTCACAGAACATGCAGAAGGTCCAAGCTATTGGGTCTATCCGAGCGGCTATCGGAGAGAGTATGCTTGAAGGTTCCTCAATGGACCGCATTAAGCGAGTCACAGAAGGACAGTTCATTCGGGAAGCCAATATGGTAACTGAGAACTATCG  >  NC_001604.1/27485‑28080`

  

Alignment Legend

Aligned base mismatch/match (shaded by quality score): `ATCG/ATCG` `< 3 ≤` `ATCG/ATCG` `< 11 ≤` `ATCG/ATCG` `< 25 ≤` `ATCG/ATCG` `< 36 ≤` `ATCG/ATCG` `< 38 ≤` `ATCG/ATCG`

Unaligned base: `atcg`    Masked matching base: `atcg`    Alignment gap: `‑`    Deleted base: `‑`
